# Genetic and environmental determinants of surface adaptations in *Pseudomonas aeruginosa*

**DOI:** 10.1099/mic.0.001335

**Published:** 2023-06-05

**Authors:** Sakthivel Ambreetha, Varsha Singh

**Affiliations:** ^1^​ Department of Developmental Biology and Genetics, Indian Institute of Science, Bengaluru, Karnataka - 560012, India

**Keywords:** biofilm, environmental signals, *Pseudomonas aeruginosa*, quorum sensing, response regulators, sensor kinases, swarming motility

## Abstract

*
Pseudomonas aeruginosa* is a well-studied Gram-negative opportunistic bacterium that thrives in markedly varied environments. It is a nutritionally versatile microbe that can colonize a host as well as exist in the environment. Unicellular, planktonic cells of *P. aeruginosa* can come together to perform a coordinated swarming movement or turn into a sessile, surface-adhered population called biofilm. These collective behaviours produce strikingly different outcomes. While swarming motility rapidly disseminates the bacterial population, biofilm collectively protects the population from environmental stresses such as heat, drought, toxic chemicals, grazing by predators, and attack by host immune cells and antibiotics. The ubiquitous nature of *P. aeruginosa* is likely to be supported by the timely transition between planktonic, swarming and biofilm lifestyles. The social behaviours of this bacteria viz biofilm and swarm modes are controlled by signals from quorum-sensing networks, LasI-LasR, RhlI-RhlR and PQS-MvfR, and several other sensory kinases and response regulators. A combination of environmental and genetic cues regulates the transition of the *P. aeruginosa* population to specific states. The current review is aimed at discussing key factors that promote physiologically distinct transitioning of the *P. aeruginosa* population.

## Introduction

*Pseudomonas aeruginosa* is a Gram-negative Gammaproteobacterium that is predominantly found in the environment, but it can also set up an infection in diverse host systems ranging from plants to animals. It is a mono-flagellated, rod-shaped bacterium that can exist in planktonic or free-living form [1]. The habitats of *P. aeruginosa* include soil, water bodies, pipelines, taps, faucets, contaminated lands, damp surfaces, contact lenses, and surgical implants [2–4]. Living organisms commonly colonized or infected by this bacterium include nematodes, insects, reptiles, plants, animals and humans [5–10]. *P. aeruginosa* is a priority level-I critical pathogen that causes terminal infections in immune-compromised individuals and patients with wound infections, chronic pulmonary disorders or surgical implants [11]. In recent decades, *P. aeruginosa* has acquired resistance against most antibiotics and is one of the leading causes of hospital-acquired infections [12]. This bacterium produces an arsenal of virulence factors, including phenazines, hydrogen cyanide, siderophores, rhamnolipids, polysaccharides, effectors of type III secretion systems, toxins, and lytic enzymes to breach the host epithelium allowing it to colonize various organs [13–17]. Several human tissues can be infected by *P. aeruginosa,* resulting in keratitis, otitis, folliculitis, osteomyelitis, pneumonia, endocarditis, urinary tract infection and wound infection [18–22].

What makes *P. aeruginosa* a successful bacterium? A large number of sensor kinases encoded in the *P. aeruginosa* genome are believed to facilitate the sensing of various cues in the environment. The enhanced sensory capability of this bacterium allows it to adopt a physiological state most conducive to its survival under any condition. The ability to switch to the biofilm state allows this bacterium to mask itself from various antibiotics and host innate immune components [23–25]. *P. aeruginosa* cells can rapidly adapt to surface conditions (stiffness or wetness) by changing flagellation status, altering pili function and modulating the release of biosurfactants [26–30]. While the flagellum is used for swimming by individual cells in a planktonic state, flagellar motility is also co-opted by a population of cells to execute swarming. However, the flagellar motion is necessary but not sufficient for swarming. *P. aeruginosa* requires both flagellation and biosurfactant (rhamnolipid) production to execute swarming on semisolid surfaces [31, 32]. On semisolid surfaces in the laboratory, believed to be mimicking mucus-covered organs, elongated and multi-flagellated swarmer cells can rapidly spread and cover the entire surface [24, 33, 34].

*P. aeruginosa* biofilm formation occurs through the aggregation of bacterial cells on moist surfaces such as stones, metal surfaces and prosthetic devices such as catheters, endotracheal tubes and contact lenses, leading to chronic infections in the urinary tract, pulmonary tract, endocardium and cornea, respectively [35–39]. The cells in a biofilm are heterogeneous [40]. Flagellated cells mediate initial adhesion to the surface and sessile cells form the core of the cell aggregate. Oxygen, as well as nutrients, are limiting in the core of the biofilm, causing altered metabolism in the inner mass of cells [41]. The biofilm population is covered with a protective mucilaginous layer made up of polysaccharides, proteins, lipids and extracellular DNA [42, 43]. *P. aeruginosa* cells predominantly produce one of the three polysaccharides, Psl, Pel and alginate, which significantly influences the biofilm architecture [44]. Psl and Pel polysaccharides are found in the non-mucoid *P. aeruginosa *, while alginate predominates in the mucoid strains [45]. Although non-mucoid and mucoid *P. aeruginosa* cells can co-exist within a biofilm, the former has higher antibiotic resistance than the latter [46]. The aggregate nature of cells in the biofilms and the extracellular matrix protects *P. aeruginosa* from hazardous chemicals, protozoan grazing, attack of antibiotics and host immune components [14, 47, 48]. There is a need to understand ways to coax the bacteria in biofilm mode to transition to a planktonic or a swarm state, so they can be targeted with existing antibiotics. However, biofilm dispersal could cause other complications in the host system. Use of biofilm-dispersing enzymes to disseminate *P. aeruginosa* cells during chronic infections can cause rapid spread of this bacteria leading to sepsis and death of the host [49]. This may result from increased virulence of the dispersing cells compared to their planktonic and biofilm counterparts [50]. Hence, co-administration of antibiotics along with a dispersal agent can reduce the survivability of the dispersed cells thereby increasing the treatment efficacy. Understanding the environmental and molecular cues promoting such transitions is the focus of this review.

Several lines of evidence indicate that biofilm formation and swarming motility are two opposing phenomena. While the *P. aeruginosa* population aggregate and produce predominantly sessile cells in the biofilm, they produce flagellated cells upon resumption of a favourable condition. Although this phenomenon is not studied systematically, it is believed that bacteria can either transition to an individual, planktonic state or a collective state of swarming and translocate to nutrient-rich locales. Other lines of evidence suggest that swarming and biofilm formation are inversely regulated in *P. aeruginosa* [33, 51]. Many two-component systems, sensor kinases, and response regulators have opposing effects on biofilm and swarming, suggesting that they likely control the switch between the biofilm and swarm states [51]. Their ligands, unknown in most cases, could be environmental cues controlling the switch between these two states of *P. aeruginosa *. Indeed, several cellular and environmental signals are known to initiate state transition in *P. aeruginosa* [52–54]. The key signals and regulatory factors associated with biofilm to swarming state transitions in *P. aeruginosa* are discussed in subsequent sections.

## Role of quorum sensing in surface adaptations of *P. aeruginosa*

**Fig. 1.**
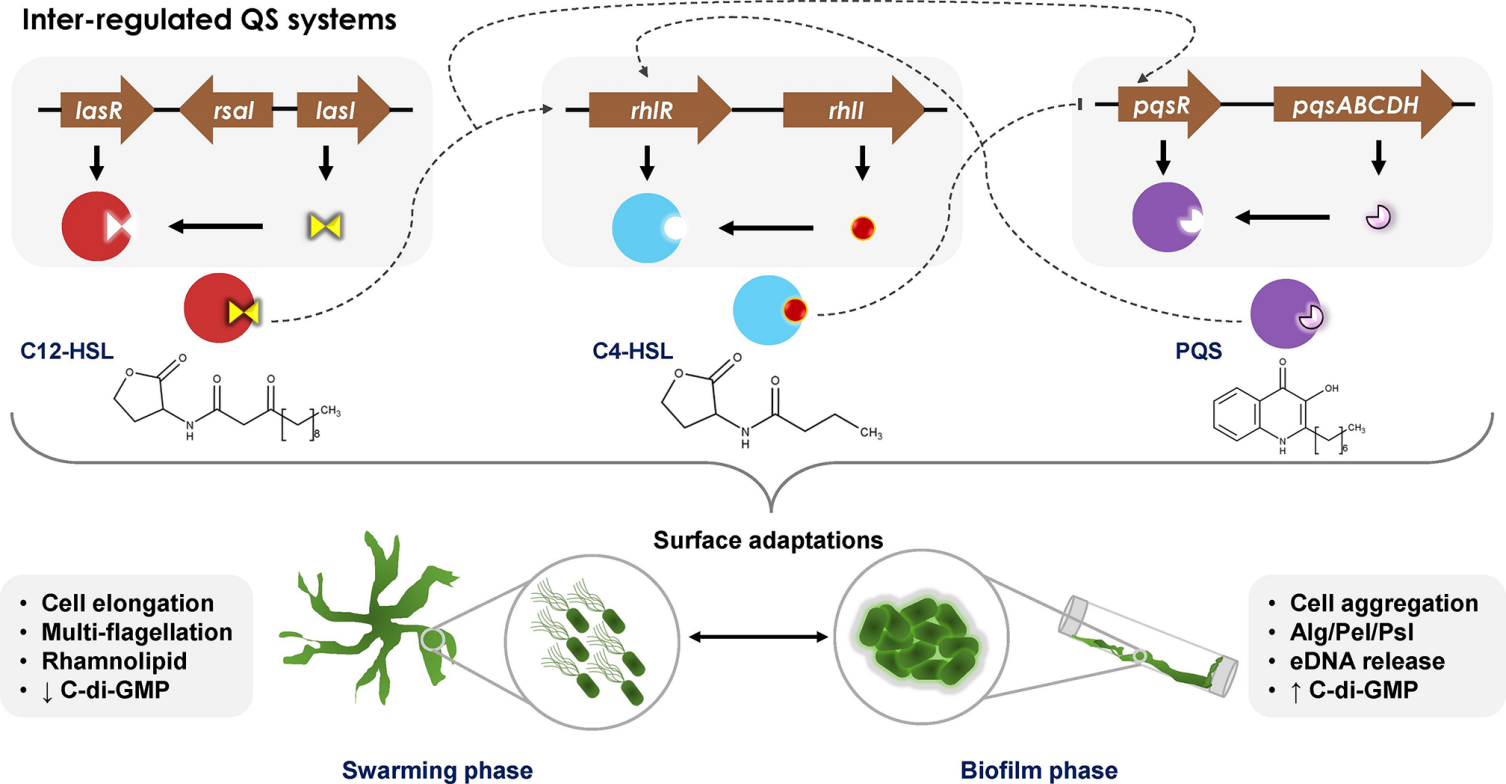
Quorum-regulated lifestyle transitions in Pseudomonas aeruginosa. Hierarchical, inter-regulated quorum sensing circuits, Las, Rhl, and Pqs produce N-(3-oxo-dodecanoyl) l-homoserine lactone (C12-HSL), N-butanoyl l-homoserinelactone (C4-HSL), and 2-heptyl-3-hydroxy-4-quinolone (PQS), respectively. Las auto-inducer activates the expression of rhl and pqs operons; Rhl auto-inducer blocks the expression of pqs operon; Pqs auto-inducer activates the expression of rhl operon. These quorum systems collectively regulate the two major surface adaptations, swarming motility and biofilm formation.

Quorum sensing (QS) is the regulation of gene expression in response to an increase in cell density. QS-sufficient bacteria produce and release chemical signals called autoinducers that increase in concentration as a function of cell density. The chemical nature of the autoinducer can vary widely from modified acyl chains to oligopeptides. The most common QS system is LuxI/LuxR, which has been identified in more than 100 Gram-negative bacterial species [55]. *P. aeruginosa* is a unique bacterium that harbours two LuxI/LuxR-type QS systems namely elastase (LasI/LasR) and rhamnolipid (RhlI/RhlR) systems. An additional non-Lux QS system named *Pseudomonas* quinolone signal (PQS) is also found in *P. aeruginosa *. These three QS circuits are inter-regulated by the auto-inducers they produce (Fig. 1) . LasI encodes an enzyme for the synthesis of 3-oxo-C12-homoserine lactone (3OC12HSL) autoinducer, which activates the transcription of *rhlR* and *pqsR* regulons [56]. RhlI encodes an enzyme catalysing the synthesis of N-butanoyl l-homoserinelactone (C4-HSL), which is a transcriptional repressor of *Pqs* QS system [57]. Conversely, 2-heptyl-3-hydroxy-4-quinolone (PQS) synthesized by the Pqs QS system turns on the expression of RhlI/RhlR operon [58, 59]. Between 5–10 % of the *P. aeruginosa* genome is regulated by QS [60, 61], suggesting that this bacterium is likely to alter its behaviour and physiology based on its strength in numbers. Regulation of rhamnolipid production, biofilm formation and motility in response to QS signals has been reported [62–64]. Rhl QS system and the rhamnolipids synthesized by this system are necessary for swarming in *P. aeruginosa* [65, 66]. Both Las and Rhl circuits are involved in biofilm differentiation and maturation [67, 68]. All the QS systems collectively regulate the biosynthesis of extracellular polysaccharides, the essential components of biofilm matrix [69, 70].

## Role of environmental factors in surface adaptations of *P. aeruginosa*

**Fig. 2.**
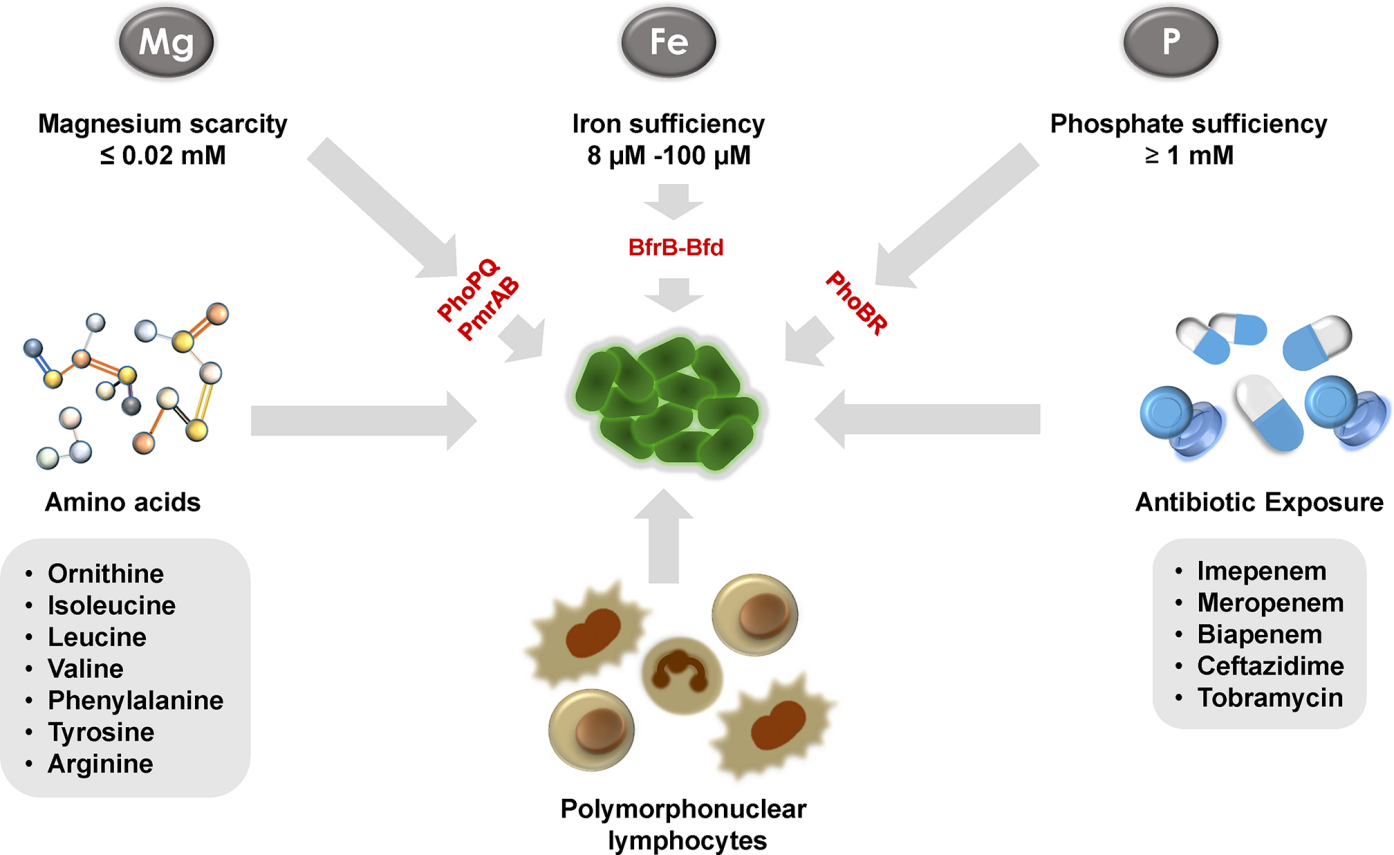
Environmental factors inducing biofilm formation in *Pseudomonas aeruginosa *. Iron and phosphate sufficiency; magnesium scarcity; exposure to specific amino acids; host immune components; and antibiotic treatment trigger planktonic to biofilm transition in *P. aeruginosa *.

Environmental factors include myriad molecules such as essential nutrients, antibiotics, secondary metabolites of neighbouring microbes and host-immune components (Fig. 2). Several environmental cues are directly or indirectly sensed by two-component signalling systems (TCS) in bacteria. Amongst the eubacteria, *Pseudomonas* genera possess a relatively larger repertoire of TCS numbering over 132 components. *P. aeruginosa* PA14 has 63 sensor kinases and 62 response regulators [71]. The first component, sensor kinase (SK) located in the plasma membrane, is phosphorylated on a specific histidine residue upon sensing a specific environmental signal. It transfers the phosphate group to a specific aspartate residue on the second component, the response regulator (RR). The RR can bring about an effect on transcription via the DNA binding domain, on cyclic nucleotide signalling via the GGDEF domain [72], or other cellular effects, which are specific to individual RRs. Several studies have analysed the effect of SK and RR mutations on biofilm formation in the endotracheal tube or the air-liquid interface in multi-well plates [51, 73]. A systematic analysis of biofilm formation and swarming in five different media (M8, M9, PGM, BM2 and M63) revealed that several TCS components regulate biofilm and swarm in an opposing manner [51]. Table 1 lists SK and RR regulating the biofilm formation or swarming motility on M9 minimal medium and their known or predicted ligands. Two TCS systems that regulate swarming in *P. aeruginosa *, PvrS/PvrR and RcsC/RcsB, are present in the highly virulent strain, PA14, but absent in the relatively less virulent strain, PAO1, suggesting that swarm-promoting TCS may have been selected for and might be linked to the virulence and survival of *P. aeruginosa *.

**Table 1.** Sensor kinases and response regulators associated with *P. aeruginosa* surface adaptations

Gene ID SK/RR		Protein SK/RR	Mutant Phenotype in M9 medium		Environmental signal	Reference
			S	B		
**PA3191**	**PA3192**	GtrS/GltR	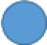	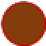	2-Ketogluconate	[51, 163]
**PA1785**	**PA1786**	NasT/NasS	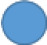	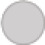	Unknown	[51]
**PA1098**	**PA1099**	FleS/FleR	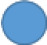	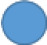	Unknown	[1, 51, 164]
**PA4197**		BfiS	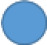	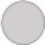	Unknown	[51, 165]
	**PA4776**	PmrA	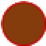	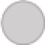	Magnesium	[51, 95, 96]
**PA3345**		HptB	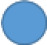	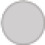	Unknown	[51, 166]
**PA2824**		SagS	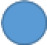	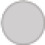	Unknown	[51]
**PA0928**	**PA2586**	GacS/GacA	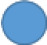	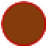	Unknown	[51, 97]
	**PA14 59770**	RcsB	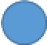	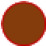	Unknown	[51, 167]
	**PA14_59790**	PvrR	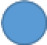	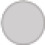	Unknown	[51, 167]
**PA4856**		RetS	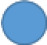	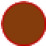	Mucin glycans	[51, 168]
**PA5484**	**PA5483**	KinB/AlgB	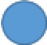	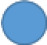	Unknown	[51, 169]
**PA0178**		CheA	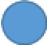	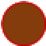	Unknown	[51]
**PA0464**		CreC	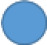	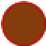	Unknown	[51]
	**PA0601**	AgtR	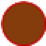	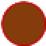	Peptidoglycan	[51, 170]
	**PA3947**	RocR	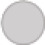	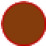	Unknown	[51]
	**PA3702**	WspR	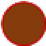	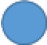	Unknown	[51, 171]
**PA3206**		CpxA	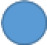	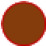	Unknown	[51, 172]
	**PA1980**	EraR	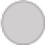	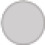	Cytosolic metabolites	[51, 173]
	**PA1799**	ParR	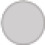	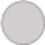	Cationic peptides	[51, 174]
	**PA3604**	ErdR	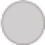	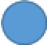	Unknown	[51, 173]
	**PA0929**	PirR	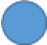	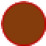	Unknown	[51]
**PA0757**		TctE	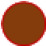	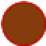	Tricarboxylic acids	[51, 175]
**PA4494**		RoxS	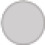	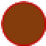	Cyanide	[51, 176]
	**PA4983**	AruR	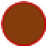	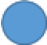	Arginine	[51, 177]
**PA5124**		NtrB	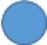	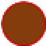	Nitrogen	[51, 178]
**PA5199**	**PA5200**	AmgS/AmgR	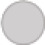	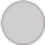	Aminoglycosides	[51, 179]
**PA5199**		EnvZ	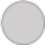	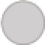	High osmolarity	[51, 180]
**PA3462**		–	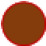	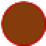	Unknown	[51]
**PA3271**		–	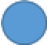	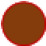	Unknown	[51]
**PA4398**		–	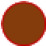	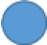	Unknown	[51]
**PA1611**		–	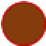	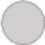	Unknown	[51]
**PA1396**		–	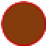	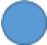	Unknown	[51]
**PA1458**		–	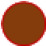	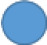	Unknown	[51]
**PA1438**		–	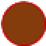	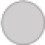	Unknown	[51]
	**PA2798**	–	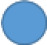	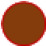	Unknown	[51]
	**PA4781**	–	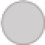	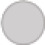	Unknown	[51]
**PA2882**		–	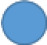	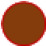	Unknown	[51]
**PA2571**		–	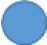	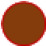	Unknown	[51]

S – swarming motility; B – biofilm formation; blue circle – weaker than wild type PA14; brown circle – stronger than wild type PA14; grey circle – similar to wild type PA14.

## Essential nutrients

Nutrient availability is one of the drivers of transitions between biofilm and swarming populations [54]. Scarcity of nutrients such as glucose and phosphates can disperse the biofilm population and trigger swarming motility in which the cells migrate along the surface in the form of tendrils to hunt for nutrients [74, 75]. Iron and phosphate limitation is common in humans with anaemia and kidney dysfunction, respectively. Such individuals are likely to be more susceptible to infection because of the ability of bacteria to disseminate through organ systems *via* swarming [24].

Iron is an essential micronutrient that facilitates redox reactions by several enzymes involved in respiration and metabolism [76]. *P. aeruginosa* and other microbes compete with the host for iron [77]. Two major iron-acquisition systems or siderophores, pyoverdine and pyochelin help *P. aeruginosa* to effectively chelate and transport iron from the extracellular environment. *P. aeruginosa* can also steal the siderophores released by other microbes. This siderophore piracy provides *P. aeruginosa* growth advantage over other microbes under nutrient-limiting conditions [78–80]. This bacterium also has bacterioferritin BfrB-Bfd complex to store iron in its insoluble form (Fe^3+^), which can be reduced and mobilized as soluble Fe^2+^ during iron scarcity [81]. Blocking the BfrB-Bfd complex alters iron homeostasis and significantly impairs biofilm development in *P. aeruginosa* [81, 82]. *P. aeruginosa* ferric uptake regulator (Fur) protein controls iron uptake and maintains cellular iron homeostasis [83, 84]. An increase in iron availability (8−100 µM) activates stable biofilm formation in *P. aeruginosa* [64, 85, 86]. On the other hand, iron concentration below 4 µM promotes swarming in *P. aeruginosa* [64, 85, 86]. Interestingly, iron limitation triggers the overproduction of rhamnolipid in *P. aeruginosa* [87]. Since rhamnolipid is essential for swarming, any condition leading to its increase will promote swarming motility in *P. aeruginosa* [62]. These lines of evidence indicate that iron availability modulates the switch between the biofilm and swarming states of *P. aeruginosa *.

Phosphate is a major macronutrient and an essential constituent of nucleic acids and phospholipids. Low phosphate (≤ 0.2 mM) in the growth medium activates the PhoB/PhoR two-component system in *P. aeruginosa* [74]. This, in turn, triggers the expression of the key regulatory genes *rhlR*, *pqsA*, *mvfR* and *lasI* in the *P. aeruginosa* QS network [88, 89]. The activation of Pho regulon via phosphate limitation or deletion of phosphate uptake protein PstS can upregulate rhamnolipid genes, which promote swarming motility and suppress the biofilm formation [90, 91]. Phosphate-limitation or hypophosphatemia is a serious condition frequently documented in patients with a kidney transplant [92]. During hypophosphatemia, the human phosphate level goes below 0.32 mM [93]. In the future, *in vitro* studies might help to identify if phosphate levels below 0.3–0.5 mM can elicit *P. aeruginosa* swarming. Moreover, clinical trials are required to check if hypophosphatemia triggers the swarming motility of *P. aeruginosa* within the host system. However, hypophosphatemia created during critical medical conditions might activate the Pho regulon, MvfR-Pqs QS pathway and pyoverdine in *P. aeruginosa* making these individuals susceptible to more severe infection by the bacterium. Although such patients might have several other health issues during such conditions, secondary infection by *P. aeruginosa* is also a serious health threat during open injuries and invasive surgeries.

Magnesium is a cofactor for bacterial metabolic enzymes and facilitates the stabilization of nucleic acids and proteins [94]. However, unlike iron and phosphate scarcity, which inhibits *P. aeruginosa* biofilms, magnesium limitation (≤ 0.02 mM) promotes biofilm formation. Magnesium scarcity activates PhoP/PhoQ and PmrA/PmrB two-component signalling systems in *P. aeruginosa* [95]. PmrB response regulator negatively regulates swarming motility in M9 minimal medium [96]. Sensor kinases also regulate biofilm formation in a magnesium-dependent manner [97]. RetS sensor kinase is a negative regulator of biofilm [98]. Magnesium limitation represses the expression of *retS,* which in turn activates *gac*, *pel* and *psl* genes in *P. aeruginosa* [99]. Moreover, the extracellular DNA present in the biofilm matrix actively chelates Mg^++^ ions sustaining magnesium-limited conditions [100]. As expected, high magnesium concentration (> 1 mM) can repress biofilm formation in *P. aeruginosa* [101]. However, the role of magnesium in swarming remains largely underexplored.

Branched-chain amino acids (isoleucine, leucine, valine) and aromatic amino acids (phenylalanine and tyrosine) can promote both biofilm formation and swarming motility of *P. aeruginosa* under *in vitro* conditions [102]. Arginine is the only amino acid known to promote *P. aeruginosa* biofilm while repressing swarming motility [102]. The effect of amino acids on the social behaviour of *P. aeruginosa* is likely mediated via 3,5-cyclic diguanylate (c-di-GMP) signalling [102]. C-di-GMP is an intracellular second messenger that gets modulated in response to environmental signals. High levels of intra-cellular c-di-GMP levels correspond to exopolysaccharide production, surface attachment and biofilm formation [103]. In contrast, low levels of c-di-GMP induce flagella- and pili-mediated surface translocations leading to swarming and twitching motilities, respectively [104].

## Host immune response

*P. aeruginosa* can breach the host epithelium and establish infection in internal organs. Invasion and colonization are aided by rhamnolipids, pyocyanin, lytic enzymes, siderophores and other virulence factors [24, 105, 106], which also activate the host’s innate immune system. Polymorphonuclear leukocytes such as macrophages, neutrophils and dendritic cells are employed for the clearance of *P. aeruginosa* in mice [107]. However, the accumulation of activated neutrophils at the site of infection causes rapid oxygen depletion and anoxia locally [108, 109]. Anoxia shuts down flagellar gene expression in this bacterium [110, 111], resulting in reduced motility [23] and initiation of a biofilm. The lack of flagellin, a major pathogen-associated molecular pattern in biofilm-forming populations of *P. aeruginosa* allows it to survive inside the host without triggering the host’s innate immune response [112]. Chronic pulmonary infections in humans are often caused by host-adapted flagellar mutants of *P. aeruginosa* that can hyper-produce exopolysaccharides [113, 114]. *P. aeruginosa* cells display several forms of adaptation inside the host. For instance, activated host neutrophils release nitric oxide, which promotes anaerobic respiration of *P. aeruginosa* cells in the biofilm [108, 115]. *P. aeruginosa* cells overproduce rhamnolipids in response to host leukocytes providing an additional layer of protection for the biofilm population [116]. Rhamnolipids produced by *P. aeruginosa* can also cause necrotic cell death in neutrophils [107]. Additionally, alginate, Psl and Pel polysaccharides present in the *P. aeruginosa* biofilm prevent opsonization by the host complement system and phagocytosis by host macrophages [117–119]. Thus, *P. aeruginosa* utilizes several signals from the host to transition between the biofilm state or the rhamnolipid-producing state.

## Antibiotics

Several intrinsic and adaptive mechanisms in *P. aeruginosa* protect it from antibiotics [120]. Switching to biofilm mode is the major adaptive response of *P. aeruginosa* cells to various classes of antibiotics. First and foremost, the biofilm layer serves as a physical barrier that limits the penetration of levofloxacin, ciprofloxacin, piperacillin, tobramycin and several other positively charged hydrophilic drugs belonging to aminoglycoside and polypeptide groups [121–125]. Moreover, antibiotics that can diffuse through the microcolonies eventually get deactivated due to the anaerobic condition in the inner layer of the biofilm structure [126, 127]. Borriello and his associates demonstrated that the 48-hour-old biofilm population of *P. aeruginosa* exhibits resistance to tobramycin, ciprofloxacin, carbenicillin, ceftazidime, chloramphenicol and tetracycline while the 4-hour-old biofilm population was susceptible to these antibiotics. The antibiotic susceptibility of the 4-hour-old biofilm population significantly decreased under anoxic conditions. This indicates that oxygen limitation can deactivate the antibiotics or *P *. aeruginosa cells growing under anoxic conditions have enhanced antibiotic resistance [128]. Furthermore, the biofilm matrix consists of antibiotic-modifying enzymes such as β-lactamases that can effectively inactivate all β-lactam antibiotics [129, 130]. Particularly, mature biofilms have been shown to produce high levels of β-lactamases when compared to young biofilms in *P. aeruginosa* [131]. Overall, the timely shift to biofilm mode helps *P. aeruginosa* to evade the host immune response and antibiotic treatments leading to persistent terminal infections in the host body. Exposure to a sub-inhibitory concentration of ciprofloxacin inhibits *P. aeruginosa* motility [132] while imipenem treatment leads to alginate hyperproduction [129]. Moreover, tobramycin-resistant biofilm was identified in *P. aeruginosa* strains isolated from cystic fibrosis patients [133]. Under *in vitro* conditions, the biofilm population of *P. aeruginosa* exhibits resistance to 100-to-1000-fold higher antibiotic concentrations than the planktonic cells [113, 134].

## Other factors controlling surface adaptations in *P. aeruginosa*

*P. aeruginosa* population often exists within poly-microbial communities inside the host and in the environment [135–137]. Thus, it is not surprising that inter-microbial interactions induce several behavioural changes in *P. aeruginosa* [138, 139]. In cystic fibrosis, *P. aeruginosa* and *Staphylococcus aureus* can co-exist while still competing for resources within the host lungs [140, 141]. In cystic fibrosis patients, dual-species biofilms of *P. aeruginosa* and *S. aureus* lead to chronic infections that coincide with the rapid deterioration of host health [142]. The spatiotemporal arrangement of *P. aeruginosa* and *S. aureus* cells within the biofilm matrix is directed by alginate, Pel, and Psl polysaccharides produced by the former bacterium [143–145]. *S. aureus* usually forms micro-colonies in the anaerobic layer while *P. aeruginosa* colonies are arranged in the aerobic layer of the biofilm matrix [146]. Non-random spatial organization of the *P. aeruginosa* and *S. aureus* populations have also been reported in chronic wound infections [147]. Poly-microbial biofilms formed by co-aggregation of *P. aeruginosa* and *S. aureus* have been detected in marketable food items as well, potentially posing a serious threat to global food safety [148]. Many other bacteria, fungi and viruses significantly influence biofilm formation in *P. aeruginosa* [149]. Respiratory syncytial virus co-infection in the host respiratory tract triggers an anti-viral immune response, which signals *P. aeruginosa* to form thick biofilms on airway epithelial cells to protect itself from the host immune attack [136]. *Streptococcus parasanguinis* and *Candida albicans* also trigger alginate–related biofilm formation in *P. aeruginosa* [150–152]. Furthermore, co-existence with *Burkholderia cenocepacia* could also boost *P. aeruginosa* biofilm development, leading to increased host inflammatory response in a mouse model system [153]. Although the molecular nature of biofilm induction is not well-understood, microbial metabolites, carbon sources or volatile cues could also induce lifestyle transitions in the *P. aeruginosa* population. For example, a low concentration of ethanol in the environment provokes *P. aeruginosa* to swarm during nutrient scarcity [154].

## Conclusion

*P. aeruginosa* has been isolated from distilled water, open ocean, oil wells, pesticide-contaminated soil, hospital sinks, water outlets, shower heads, flowers, fruits, vegetables, nematodes, insects, reptiles, animals, humans, surgical tools and medical implants in different parts of the world [3, 7–9, 155–159]. This bacterium is identified in most environments associated with anthropogenic activities [160]. The ecological success of *P. aeruginosa* is linked to its ability to adapt by acquiring new genetic elements and losing non-essential genes [161], its metabolic plasticity [162], and its ability to produce virulence factors and secretory molecules such as phenazines, lytic enzymes (elastase, protease, lipase), rhamnolipids, exotoxin A, alginate, pyoverdine and pyochelin [13]. In addition, its ability to switch between the motile swarming phase and the sessile biofilm phase is also an important adaptation. Identifying and understanding signals that coax this bacterium to transition from the antibiotic resistant (biofilm) state to the antibiotic-susceptible (planktonic or swarming) states might be the holy grail to treat *P. aeruginosa* associated infection
